# Rural-urban differences in pain intensity, pain interference, and physical function among cancer survivors

**DOI:** 10.21203/rs.3.rs-10832930/v1

**Published:** 2026-10-14

**Authors:** Gaurav Kumar, Motolani E. Ogunsanya, Amy M. Cohn, Deeksha Gupta, Nathalia M. Machado, Brittany L. Hudson, Munjireen Sifat, Christina Henson, Susanna Ulahannan, Darla E. Kendzor

## Abstract

Rural cancer survivors may face structural barriers to symptom management and rehabilitation, yet rural-urban differences in pain and physical function remain understudied. This secondary analysis examined rural-urban differences in pain intensity, pain interference, and physical function among 232 adults receiving care at a National Cancer Institute-designated cancer center in Oklahoma. Rural-urban residence was classified using 2020 Rural-Urban Commuting Area codes based on residential ZIP code. Outcomes were assessed using the Patient-Reported Outcomes Measurement Information System-29. Multivariable linear regression and mixed-effects models examined baseline and longitudinal associations, adjusting for sociodemographic and clinical covariates. Among participants, 92 (39.7%) were rural and 140 (60.3%) were urban. Compared with urban participants, rural participants reported higher baseline pain intensity (4.3 vs 3.2; p = 0.003), higher pain interference (57.3 vs 53.6; p = 0.011), and lower physical function (41.8 vs 44.4; p = 0.034). In longitudinal models, rural residence remained associated with higher pain intensity (β = 0.99; p = 0.006), higher pain interference (β = 3.45; p = 0.018), and lower physical function (β = -2.51; p = 0.048). Findings support targeted pain management, rehabilitation, and virtual care strategies for rural cancer survivors.

